# Insulin BBB pharmacokinetics in young apoE male and female transgenic mice

**DOI:** 10.1371/journal.pone.0228455

**Published:** 2020-01-31

**Authors:** Elizabeth M. Rhea, Eileen Ruth S. Torres, Jacob Raber, William A. Banks

**Affiliations:** 1 Division of Gerontology and Geriatric Medicine, Department of Medicine, University of Washington, Seattle, Washington, United States of America; 2 Research and Development, Veterans Affairs Puget Sound Health Care System, Seattle, Washington, United States of America; 3 Department of Behavioral Neuroscience, Oregon Health & Science University, Portland, Oregon, United States of America; 4 Division of Neuroscience, Departments of Neurology and Radiation Medicine, ONPRC, Oregon Health & Science University, Portland, Oregon, United States of America; 5 Geriatric Research Education and Clinical Center, Veterans Affairs Puget Sound Health Care System, Seattle, Washington, United States of America; Hungarian Academy of Sciences, HUNGARY

## Abstract

In addition to age, apolipoprotein E4 (E4), female sex, or a combination of both synergistically increase the risk for the development of Alzheimer’s disease (AD). Why these risk factors predispose an individual to developing AD later in life is the target of the current investigation. Central nervous system (CNS) insulin resistance is associated with cognitive impairment and AD. CNS insulin is acquired primarily from the circulation and therefore must negotiate the blood-brain barrier (BBB). Thus, changes in BBB transport of insulin could lead to alterations in CNS insulin signaling and resistance, which would then lead to changes in cognition. There has been recent evidence suggesting the relationship between CNS insulin; E4, a risk factor to develop AD as compared to E3; and the female sex in aged individuals and in pre-clinical models. However, this relationship has been largely unexplored at a younger age, in which some of these risk factors could predispose an individual to dysregulation of CNS insulin later in life. Here, we present the first findings of BBB insulin pharmacokinetics in young E3 and E4 male and female targeted replacement (TR) mice. We found that levels of insulin binding the vasculature at the BBB are different due to genotype and sex which could impact the function of the brain endothelial cell. These early alterations could contribute to or fully explain the age-related cognitive changes observed due to CNS insulin signaling in E4 and/or female individuals.

## Introduction

Impairments in the regulation of central nervous system (CNS) insulin are clearly associated with age-related cognitive decline (ACD), mild cognitive impairment (MCI), and Alzheimer’s disease (AD). Although age is the greatest risk factor for developing ACD, there are many other risk factors involved that can accelerate this decline, including apolipoprotein E4 (E4) as compared to apoE3 (E3), and female sex or a combination of both synergistically increasing AD risk [1]. However, it remains unclear why these features are implicated in ACD. One common thread between these risk factors is insulin resistance in the CNS, defined broadly in this context as impaired insulin activity in the CNS.

Insulin in the CNS is primarily derived from blood insulin and is transported across the blood-brain barrier (BBB) [2–4]. Insulin can also act directly on the BBB to alter transport of other important nutrients, including amino acids, into the CNS [5, 6]. CNS insulin action is impaired in AD [7] and this insulin deficiency is more evident in those with advanced AD or E4 carriers without advanced AD [8, 9]. These results point to a disruption in brain insulin metabolism in AD as well as in non-demented E4 carriers.

Indeed, the E4 isoform doubles the risk for developing AD [10–13] and genome-wide association studies confirm that E4 is the most potent genetic risk factor for developing AD [14, 15]. The fact that environmental factors like a healthy lifestyle predominantly protect non-E4 carriers might contribute to the E4 risk [16]. Female sex is also a risk factor for developing MCI and AD [1, 17]. The two risk factors can work synergistically to further increase the risk for cognitive impairment and cognitive decline [18, 19]. Consistent with the human findings, female mice expressing E4 in the brain are more susceptible to cognitive impairments than female mice expressing E3 or male mice expressing E4 [20–23]. In E4 mice, these impairments occur by 6 months of age in female mice, yet by 18 months of age, male mice are still cognitively intact [22].

Insulin sensitivity is part of the developmental origin of health and disease hypothesis. In this study, we started to investigate whether deficits in CNS insulin signaling in adult E4 and/or female mice arise due to changes that occur early and thus, predispose mice to impairments at a later age. One of the mechanisms by which deficits in CNS insulin signaling could arise is by impaired insulin transport at the BBB. Therefore, we analyzed and compared insulin BBB pharmacokinetics in young, 2–4 month old E3 and E4 male and female mice.

## Materials and methods

### Animals

Female and male homozygous human E3- and E4-targeted replacement (TR) mice, generated as described [24, 25], were bred in house at the Oregon Health & Sciences University (OHSU) prior to transfer to the Veterans Affairs Puget Sound Health Care System (VAPSHCS). Mice had *ad libitum* access to food and water and were kept on a 12/12 hour light/dark cycle. Mice were 2–4 months of age on the day of the study, with E4 mice approximately 1–2 months younger than E3 mice. All procedures complied with the National Institutes of Health Guide for the Care and Use of Laboratory Animals and with local Institutional Animal Care and Use Committee (IACUC) approval at both OHSU (Protocol Number: 00000262) and the VAPSHCS (Protocol Number: 0936). The mice were bred and the study was performed at facilities approved by the Association for Assessment and Accreditation of Laboratory Animal Care International (AAALAC). All surgery was performed under urethane anesthesia and all efforts were made to minimize discomfort.

### Radioactive labeling of insulin and albumin

Human insulin (10 μg) (Sigma-Aldrich, St. Louis, MO, USA) was diluted in 0.25 M chloride-free sodium phosphate buffer (PB), pH 7.5, and radioactivity labeled with 0.5 mCi Na^125^I (Perkin Elmer, Waltham, MA, USA) by the chloramine-T (Sigma-Aldrich) method. Chloramine-T (10 μg) was diluted in 0.25 M PB (10 μL) and added to the insulin solution to begin the reaction. The reaction was terminated 1 min later with the addition of 100 μg sodium metabisulfite diluted in 0.25 M PB (10 μL). Bovine serum albumin (BSA, Sigma-Aldrich) was radioactively labelled with ^99m^Tc (GE Healthcare, Seattle, WA, USA). Briefly, 1 mg albumin was combined with 120 μg stannous tartrate and 20 μl 1 M HCl in 500 mL deionized water. For the 20 min reaction, 1 mCi of ^99m^Tc was added. ^125^I-insulin and ^99m^Tc-albumin were purified on a column of Sephadex G-10 (Sigma-Aldrich). Protein labelling by ^125^I or by ^99m^Tc isotopes was characterized by 15% trichloroacetic acid (TCA) precipitation. Greater than 90% radioactivity in the precipitated fraction was consistently observed for insulin and albumin.

### BBB pharmacokinetic transport of iodinated insulin

Mice were anesthetized with an intraperitoneal injection of 0.1 mL 40% urethane to minimize pain and distress. Mice received a bolus injection into the right jugular vein of 0.2 mL of 1% BSA/lactated Ringer’s solution (LR) containing 1x10^6^ cpm of ^125^I-insulin and 5x10^5^ cpm of ^99m^Tc-albumin. The injection volume was chosen based on historical experiments with mice of similar size, which is not estimated to significantly affect overall blood volume, yet still large enough to allow for accuracy of the injected solution. We correct for the exact amount of radioactivity injected based on serum levels. The estimated amount of ^125^I-insulin injected is approximately 10 ng/mL, a dose which does not affect blood glucose [2]. ^99m^Tc-albumin was co-injected as a marker for vascular space [26]. Blood from the left carotid artery was collected between 0.5–10 min after intravenous injection. Mice were immediately decapitated and their whole brains quickly removed and weighed. The arterial blood was centrifuged at 5400*g* for 10 min at 4°C and serum collected. The brains were dissected into regions according to the method of [27] and weighed. The levels of radioactivity in serum (50 μL) and brain regions were counted in a gamma counter (Wizard2, Perkin Elmer, Waltham, MA). The brain/serum (B/S) ratios were graphically displayed against their respective exposure times (Expt). Expt was calculated from the formula:

$$Exposure\;time=\frac{\int_{0}^{t}Cp(t)dt}{Cp(t)}$$ (1)

where *Cp* is the level of radioactivity (cpm) in serum at time (*t*). Expt corrects for the clearance of peptide from the blood. The clearance of ^125^I-insulin was calculated using the inverse slope of the linear portion of the log base 10 serum ^125^I-insulin levels over time, multiplied by 0.301. The influx of insulin was calculated by multiple-time regression analysis as described by Patlak, Blasberg, and Fenstermacher [26, 28]:

$$\frac{Am}{Cpt}=Ki(\frac{\int_{0}^{t}Cp(t)dt}{Cp(t)})+Vi$$ (2)

where *Am* is level of radioactivity (cpm) per g of brain tissue at time *t*, *Cpt* is the level of radioactivity (cpm) per mL arterial serum at time *t*, *K*_i_ (μL/g-min) is the unidirectional solute influx from blood to brain, and *V*_i_ (μL/g) is the level of rapid and reversible binding for the brain vasculature. The brain/serum (B/S) ratios for insulin were corrected for vascular space by subtracting the corresponding ratio for albumin, yielding a delta B/S ratio. The linear portion of the relation between the delta B/S ratio versus Expt was used to calculate the *K*_i_ (μL/g-min) with its standard error term, and the y-intercept determined as representation of the *V*_i_ (μL/g) for each brain region [26]. For whole brain, the weights and cpm of all individual brain regions except for the olfactory bulb were summed to calculate the B/S ratio. As the B/S ratio for ^99m^Tc-albumin did not differ with time, the vascular space for each genotype and sex was also calculated by collapsing values across time within each brain region. All anesthetized mice were killed by decapitation at the end of the study.

### Statistics

Regression analysis and other statistical analyses were performed with the use of Prism 8.0 (GraphPad Software Inc., San Diego, CA, USA). Brain weight means are reported with their standard error terms and compared by two-way analysis of variance (ANOVA) followed by Sidak’s post hoc test to determine differences due to genotype or sex. All analyses passed the Shapiro-Wilk normality test. For pharmacokinetic studies (multiple-time regression analysis), the slope of the linear regression lines (*K*_*i*_), reported with their correlation coefficients (*r*), and y-intercepts (*V*_*i*_) were compared statistically with the Prism 8.0 software package, as described [29].

## Results

### Brain weights

**Fig 1:**
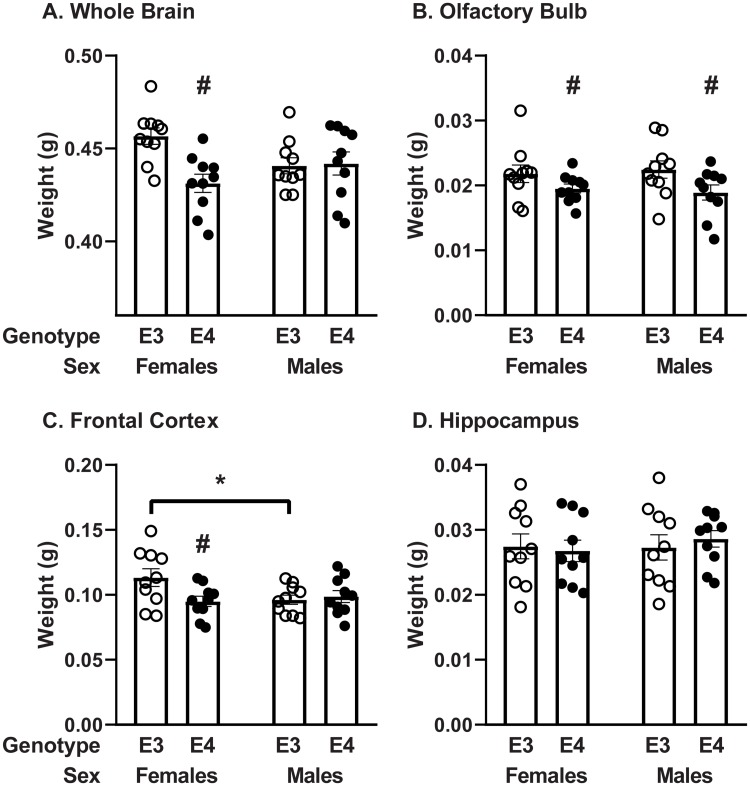
Effect of apoE genotype and sex on brain region weights. A) Whole brain, B) olfactory bulb, C) frontal cortex, and D) hippocampus weights. Data are presented as means ± SEM. *n* = 10 mice/group. ^#^ *p* < 0.05, E3 vs E4; Interaction * *p* < 0.05 Male vs Female; two-way ANOVA followed by Sidak’s multiple comparison test.

There was a significant interaction between sex and genotype in the weight of the whole brain (Fig 1A, *p* = 0.012, *F* (1, 36) = 7.03). In addition, whole brain weights differed due to genotype (*p* = 0.021, *F* (1, 36) = 5.80). The brain weights for every region dissected were also recorded. The olfactory bulb weighed significantly less in E4 than E3 mice, regardless of sex (Fig 1B, *p* = 0.018, *F* (1, 36) = 6.16). The olfactory bulb serves at least two critical functions in mice: olfaction and memory. In addition, for the frontal cortex, another region important in memory formation, there was a significant interaction between sex and genotype (Fig 1C, *p* = 0.040, *F* (1, 36) = 2.65). The frontal cortex weight in the E3 females was approximately 15% greater than that in E4 females or E3 males (*p* < 0.05). The hippocampus did not differ in weight among the groups (Fig 1D). The weights of the remaining regions of the brain were not different between genotypes or sexes.

### Brain vascular space

Brain vascular space was measured by co-injecting ^99m^Tc-albumin. This marker is used to measure the amount of vascular space within each region in order to correct brain ^125^I-insulin levels. Since values did not change with time, ^99m^Tc-albumin values were collapsed to quantify the average vascular space within each region (Table 1). These levels are independent from cerebral blood flow, which requires other methods for measurement. There were no differences in regional vascular space due to genotype or sex. Vascular space did significantly vary regionally, with the olfactory bulb and pons/medulla having the highest (mean = 11.24 ± 1.12 and 10.70 ± 0.65 μL/g, respectively) and the striatum and thalamus having the lowest value (mean = 3.94 ± 0.81 and 5.22 ± 0.37 μL/g-min, respectively), as has been previously shown for mice [30].

**Table 1:** Vascular space.

Group	E3 Female		E3 Male		E4 Female		E4 Male	
Region	Mean	SE	Mean	SE	Mean	SE	Mean	SE
**Whole Brain**	6.75	0.32	6.87	0.17	6.67	0.31	6.95	0.24
**Olfactory Bulb**	10.63	1.24	11.37	0.86	10.71	1.05	12.23	1.32
**Striatum**	4.49	0.41	4.42	0.27	4.10	0.53	5.40	0.65
**Frontal Cortex**	5.41	0.41	5.62	0.38	5.21	0.71	5.48	0.49
**Hypothalamus**	5.79	0.86	6.18	0.85	6.48	0.85	5.05	0.91
**Hippocampus**	6.72	0.68	6.10	0.60	6.14	0.50	6.22	0.63
**Thalamus**	4.46	0.60	5.19	0.31	5.67	0.25	5.55	0.29
**Parietal Cortex**	6.22	0.44	5.78	0.26	6.31	0.52	6.68	0.53
**Occipital Cortex**	7.51	0.72	7.16	0.43	6.74	0.17	6.51	0.50
**Cerebellum**	8.66	0.44	9.36	0.48	8.49	0.69	9.64	0.45
**Midbrain**	7.09	0.53	6.30	0.47	6.75	0.42	6.81	0.48
**Pons/Medulla**	10.99	0.56	10.67	0.57	10.20	0.69	10.92	0.78

Vascular space as represented by the level of Tc99m-albumin present in each brain region (g) in relation to serum levels (μL). Average values (mean) are taken across time and represented as μL/g +/- SE. *n* = 8–10 mice/group.

### ^125^I-insulin serum clearance

**Fig 2:**
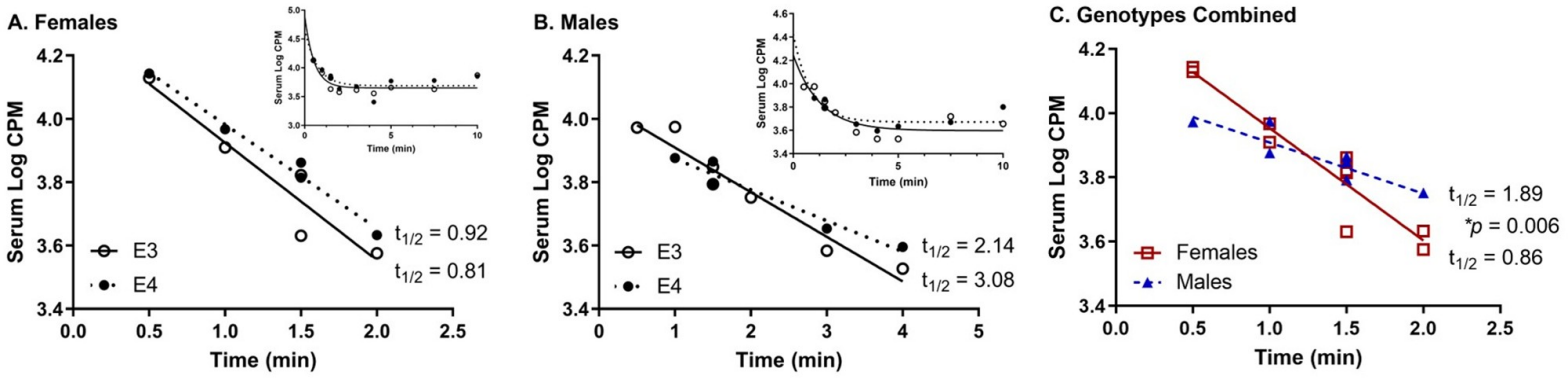
Serum clearance (linear) of ^125^I-insulin in E3 and E4 mice. There were no differences between genotypes in A) female and B) male mice. Due to lack of differences between genotypes, C) genotypes are combined within each sex. Linear regression analysis reveals a significant difference in the clearance rate between females and males (* *p* = 0.006). Insets show entire clearance curve.

The serum clearance of ^125^I-insulin was analyzed in each group (Fig 2). There was no difference in the clearance between genotypes (Fig 2A and 2B). Therefore, genotypes were collapsed to determine if there were sex differences (Fig 2C). Males had over a 2-fold slower clearance rate compared to females (*p* < 0.01, *F* = 10.43).

### ^125^I-insulin BBB pharmacokinetics

**Fig 3:**
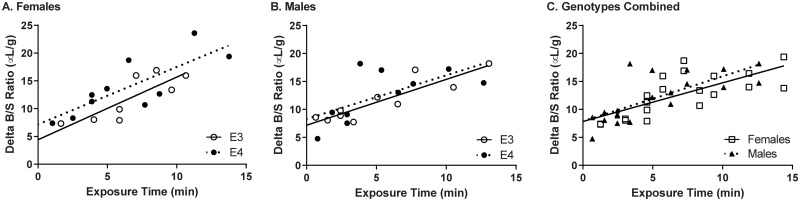
Transport of ^125^I-insulin across the BBB in E3 and E4 mice. There were no differences in the transport rate between genotypes in A) female and B) male mice. C) Genotypes are combined within each sex. Linear regression analysis computes the transport rate (*K*_i_) and reversible vascular binding (*V*_i_) for each group.

^125^I-insulin transport into the whole brain occurred in all groups. There was no significant difference in the transport rate (*K*_i_) due to genotype or sex (Fig 3). The corresponding pharmacokinetics for ^125^I-insulin into whole brain is indicated in Table 2. The transport rate for each brain region is indicated in Table 3. There were some regions where ^125^I-insulin transport did not occur (listed as “ns” for non-significant). There were no differences in transport rate due to genotype or sex in any brain region.

**Table 2:** Pharmacokinetics of ^125^I-insulin at the BBB in whole brain.

Sex	Genotype	*K*_i_ (μL/g-min)	*r*	*V*_i_ (uL/g)
Female	E3	1.128 ± 0.32	0.82	4.41 ± 2.31
	E4	1.033 ± 0.28	0.80	7.17 ± 2.07
Male	E3	0.820 ± 0.14	0.90	7.17 ± 0.95
	E4	0.781 ± 0.32	0.65	8.23 ± 2.13
Female	Combined	0.691 ± 0.16	0.72	7.84 ± 1.26
Male	Combined	0.803 ± 0.17	0.74	7.88 ± 1.09

Values are generated from the multiple-time regression analysis.

**Table 3:** Insulin transport rate within each brain region.

Group	E3 Female		E3 Male		E4 Female		E4 Male	
Region	Mean	SE	Mean	SE	Mean	SE	Mean	SE
Olfactory Bulb	2.178	0.67	1.607	0.68	1.530	0.44	1.712	0.71
Striatum	ns		ns		ns		ns	
Frontal Cortex	0.796	0.29	0.895	0.10	0.720	0.17	1.023	0.24
Hypothalamus	1.541	0.61	1.425	0.35	1.116	0.41	2.272	0.42
Hippocampus	ns		1.813	0.54	1.171	0.47	ns	
Thalamus	0.752	0.32	ns		0.634	0.20	1.203	0.35
Parietal Cortex	0.550	0.22	0.961	0.13	0.859	0.23	0.920	0.25
Occipital Cortex	0.742	0.23	0.826	0.18	0.811	0.28	0.793	0.23
Cerebellum	1.189	0.46	1.159	0.31	2.110	0.69	ns	
Midbrain	0.689	0.26	1.244	0.34	0.699	0.15	0.887	0.37
Pons/Medulla	1.462	0.35	0.739	0.16	1.111	0.40	ns	

Data represents the *K*_i_ (μL/g/min) +/- SEM. *n* = 7–10 mice/group; ns = non-significant transport

**Fig 4:**
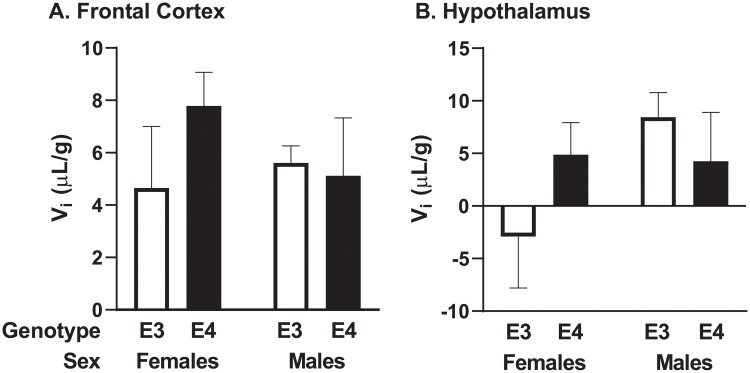
Reversible ^125^I-insulin vascular binding (*V*_i_). Graphical representation of the A) Frontal cortex and B) hypothalamus vascular binding. Data is presented as mean ± SEM; *n* = 8–10 mice/group. ANCOVA analysis: *p* = 0.039, *F* = 3.113 and *p* < 0.0001, *F* = 10.76, respectively for each brain region.

The amount of reversible vascular binding (*V*_i_) for ^125^I-insulin in each brain region is indicated in Table 4. In regions where transport did not occur (Table 3, ns), the amount of reversible binding is not able to be measured (Table 4, nm: non-measurable). Regions that had statistical differences in the amount of reversible binding are graphed in Fig 4 (frontal cortex: *p* = 0.039, *F* = 3.113 and hypothalamus: *p* < 0.0001, *F* = 10.76). This suggests subtle differences in the uptake and/or binding of insulin at this early age.

**Table 4:** Reversible ^125^I-insulin vascular binding (*V*_i_) within each brain region.

Group	E3 Female		E3 Male		E4 Female		E4 Male	
Region	Mean	SE	Mean	SE	Mean	SE	Mean	SE
Olfactory Bulb	6.67	5.42	11.89	4.48	11.71	3.25	13.31	4.6
Striatum	nm		nm		nm		nm	
Frontal Cortex*	4.65	2.35	5.61	0.65	7.79	1.28	5.90	1.33
Hypothalamus*	-2.91	4.90	8.45	2.33	4.88	3.04	5.63	2.28
Hippocampus	nm		11.37	2.38	10.01	3.52	nm	
Thalamus	4.04	2.89	nm		7.60	1.48	4.56	2.3
Parietal Cortex	4.50	2.02	4.17	0.88	3.31	1.74	4.74	1.6
Occipital Cortex	6.73	1.68	5.50	1.21	6.53	2.12	6.71	1.6
Cerebellum	8.36	3.71	8.90	2.06	8.91	3.50	nm	
Midbrain	4.41	2.37	4.81	1.36	5.93	1.12	6.38	2.4
Pons/Medulla	3.90	2.53	7.70	1.04	8.76	3.02	nm	

Data represents the *V*_i_ (μL/g) +/- SE. *n* = 7–10 mice/group. nm = non-measurable

## Discussion

This is the first study to investigate the pharmacokinetics of insulin transport across the BBB in E3 and E4 male and female mice. We found apoE genotype differences in the weight of the olfactory bulb and total brain weight. There were no differences in the amount of vascular space within each brain region between the groups. Serum clearance of ^125^I-insulin was slower in males compared to females. Lastly, while there was no difference in the rate of ^125^I-insulin transport across the BBB into brain, there were regional differences in the amount of ^125^I-insulin vascular binding as measured by *V*_i_. These findings suggest there may be BBB changes early on regarding apoE genotype and sex.

The whole brain weight was significantly decreased in E4 female mice compared to E3 female mice. This decrease was primarily driven by decreases in the frontal cortex. In addition, the olfactory bulb weight was significantly lower in E4 than E3 mice, regardless of sex. There were no other regional differences in brain weight due to genotype or sex. While the mice in this study were all within 2 months of age at the time of the study, the E4 mice were a little younger than the E3 mice. Therefore, based on this study, it is difficult to conclude whether the decreased brain weight is due to genetic differences or solely based on age. While this age difference does not affect our primary outcome (insulin transport rate), future studies will be executed on mice of more similar ages.

Despite this difference in weight, there were no differences in vascular space, suggesting the weight differences are due to parenchymal changes. The amount of vascular space per weight of tissue could indicate early anatomical changes that leads to memory changes later on, especially following environmental challenges. Young E4 mice have been shown to exhibit mild cognitive impairment compared to E3 mice using different learning and memory tests [31–33]. In older mice (15-month-old), there are decreases in cerebral blood volume in the pial vessel branches of the middle cerebral artery of female E4 mice [34].

Serum clearance of insulin is predominantly affected by tissue uptake [35]. While there were no genotype differences in the serum clearance rate of ^125^I-insulin, there was a sex difference. Females had over a two-fold faster rate in clearance compared to males. Female adolescents have an increased insulin clearance rate compared to males [36]. Despite the faster clearance rate for insulin in females, it is unlikely that this contributes to CNS insulin levels. CNS insulin uptake is saturable and based on need rather than concentration gradient flow. Unlike glucose uptake that is impacted by flow rate, the rate of insulin transport is independent of cerebral blood flow [2, 5].

Our studies are designed to investigate the transfer of human ^125^I-insulin from the periphery to the CNS. Rat and human insulin are transported across the BBB at similar rates and are both saturable by excess respective peptide [2]. For this study, we wanted to define the transport rate of ^125^I-insulin across the BBB in young E3 and E4 male and female mice to determine if there were changes that could predispose these mice to cognitive changes. We used the well-described multiple-time regression analysis technique first described by Patlak, Blasberg, and Fenstermacher [26, 28]. This analysis takes into consideration the amount of radioactivity present in the periphery and CNS, and therefore, the rate of transfer is for the BBB. Insulin does bind to brain but brain is one of the compartments (CNS) used in the analysis. There were no differences in the transport rate in any of the groups, for any of the brain regions investigated. The whole brain transport rates ranged from 0.781 ± 0.32 μL/g-min in E4 males to 1.128 ± 0.32 μL/g-min in E3 females. These values are similar to the average rate that we have previously observed in mice on a similar background, 0.71 ± 0.17 μL/g-min [37]. Insulin, like other peptides, binds minimally to albumin. Furthermore, these interactions are weak, so receptors and transporters dominate insulin action and pharmacokinetics [38].

Lastly, we compared the amount of reversible vascular binding of ^125^I-insulin. Multiple-time regression analysis divides kinetics into a measure of *K*_i_, the rate of transport into brain, and *V*_i_, the reversible binding to the vasculature. There were two regions that had significant differences in *V*_i_ between the groups. In the frontal cortex, E3 females had the lowest level (4.65 ± 2.4 μL/g) while E4 females had the highest (7.79 ± 1.3 μL/g) amount of reversible binding. In the hypothalamus, E3 females again had the lowest (-2.91 ± 4.9 μL/g), but E3 males had the highest (8.45 ± 2.3 μL/g). This could suggest that there is a change in the amount of available insulin binding sites at the luminal surface of the brain endothelial cell in these regions, since brain endothelial cells primarily comprise the surface of the vasculature. Insulin has several effects on brain endothelial cell function, including affecting the transport of amino acids into the brain which are important for monoamine and kynurenine brain levels [39, 40]. Whether or not the increased insulin vasculature binding in brains of E4 mice compared to those in E3 mice in these regions leads to alterations later in life remains to be determined. Brain insulin binding sites in rats vary regionally, with the olfactory bulb containing the highest amount, the cerebral cortex and hippocampus the next highest amount, and the pons/medulla containing the least as measured by ^125^I-insulin binding experiments [41]. The expression of the insulin receptor protein using an antibody directed towards the β subunit of the receptor is highest in the olfactory bulb, thalamus, and hippocampus [42]. However, it was also determined mRNA did not match with protein expression in all regions, suggesting different requirements for the receptor [42]. We have found insulin transport across the BBB can occur independently of the insulin receptor [37].

It should be noted that our studies do not differentiate between the amount of ^125^I-insulin present inside the brain capillary versus complete transcytosis into the brain parenchyma. However, previous studies designed to investigate the transport of ^125^I-insulin have shown that the majority of ^125^I-insulin enters the brain parenchyma [37], using the well-described capillary depletion method to determine distribution between these two compartments [43].

In summary, there are multiple lines of evidence that there are alterations in the brain of young mice in regards to apoE genotype and sex. First, we found differences in the size of the olfactory bulb and frontal cortex that could be an important indicator of memory. Second, we found differences in insulin BBB pharmacokinetics due to apoE genotype and sex, with E4 mice having greater insulin binding to brain vasculature than E3 mice. These findings suggest that CNS changes in the context of tissue weight and insulin BBB pharmacokinetics could be occurring earlier in life that might lead to altered cognition later in life.
